# Investigation on the antimicrobial properties of cerium‐doped bioactive glasses

**DOI:** 10.1002/jbm.a.37289

**Published:** 2021-08-04

**Authors:** Stefano Raimondi, Alfonso Zambon, Raffaella Ranieri, Francesca Fraulini, Alberto Amaretti, Maddalena Rossi, Gigliola Lusvardi

**Affiliations:** ^1^ Department of Life Sciences University of Modena and Reggio Emilia Modena Italy; ^2^ Department of Chemistry and Geological Sciences University of Modena and Reggio Emilia Modena Italy; ^3^ Biogest‐Siteia University of Modena and Reggio Emilia Reggio Emilia Italy

**Keywords:** antimicrobial activity, bioactive glasses, cerium

## Abstract

Cerium‐doped bioactive glasses (Ce‐BGs) are implant materials that present high biocompatibility, modulate the levels of reactive oxygen species, and exert antimicrobial activity. The potential of BGs, 45S5, and K50S derived glasses doped with CeO_2_ (1.2, 3.6, and 5.3 mol%) to inhibit the growth of pathogen microbes was thoroughly investigated according to the ISO 22196:2011 method properly adapted. A significant reduction of the *E. coli* charge was detected in all glasses, including the BGs without cerium. The evolution of pH of the medium not inoculated following the immersion of the Ce‐BGs was monitored. The presence of cerium did not affect markedly the pH trend, which increased rapidly for both compositions. The change of pH was strongly mitigated by the presence of 200 mM phosphate buffer pH 7.0 (PB) in the medium. In media buffered by PB, the growth of *E. coli*, *Pseudomonas aeruginosa*, *Listeria monocytogenes*, *Staphylococcus aureus*, and *C. albicans* was not affected by the presence of BGs doped or not with cerium, suggesting that the antibacterial activity of Ce‐BGs is linked to the increase of environmental pH rather than to specific ion effects. However, Ce‐BGs resulted promising biomaterials that associate low toxicity to normal cells to a considerable antimicrobial effect, albeit the latter is not directly associated with the presence of cerium.

## 1 INTRODUCTION

Bioactive glasses (BGs) are implant materials that can be used for biomedical applications, such as dentistry, orthopedics, and maxillofacial surgery. BGs present high biocompatibility and can effectively promote bone and soft tissue regeneration.^1^ Phospho‐silica‐based 45S5 Bioglass®^2^, ^3^ (abbreviated as 45S5) and silica‐based Kokubo glass (abbreviated as K50S)^4^ are among the earliest developed and best characterized BGs and show comparable bioactivity. The properties of BGs can be improved by doping with therapeutic inorganic ions (TII)^5^; the addition of cerium to 45S5 (H series) and to K50S (K series) allowed us to obtain novel cerium‐containing bioactive glasses (Ce‐BGs) with improved cytocompatibility and antioxidant properties.^6^, ^7^, ^8^, ^9^, ^10^, ^11^

Cerium is the first element in the lanthanide group, and it is the only lanthanide stable in the tetravalent state. The easy exchange between Ce^3+^ ↔ Ce^4+^ oxidation states underlies its catalytic activity as a scavenger of reactive oxygen species (ROS), and thus its antioxidative properties that protect osteoblasts from oxidative stress.^12^ Furthermore, Ce‐BGs are nontoxic to the cells and enhance the osteoblastic differentiation, the mineralization of primary osteoblasts, and the production of collagen.^13^ Our studies on H and K cerium doped series show that the presence of cerium enhances proliferation and vitality of osteocyte‐like cells.^7^, ^8^, ^14^ We have also examined the structural role of cerium in the BGs; in the K series, cerium is coordinated by non‐bridging oxygens (NBOs), whereas in the H series, the NBOs around cerium ions belong to orthophosphate groups. The latter groups stabilize the Ce^3+^ ions subtracting them from the interconversion process between Ce^3+^ and Ce^4+^; this could explain the higher catalase mimetic activity of the K with respect to the H series.^10^

Importantly, the efficacy of a BG in inducing bone regeneration requires the prevention of bacterial adhesion and proliferation that can occur on the implant surface.^15^ Antibacterial properties of BGs can be induced or improved by the addition of metal ions with bactericidal effects. BGs doped with silver, copper, zinc, and gallium are considered potential candidates as antibacterial agents.^16^, ^17^, ^18^, ^19^, ^20^, ^21^, ^22^, ^23^, ^24^

Cerium salts (oxide, nitrate, chloride, etc.) were among the first agents used against bacterial species, with evidence of some antibacterial activity dating back to 1947.^25^ Cerium ions bind rapidly to *E. coli* cells, interfering with respiration and other metabolic functions.^26^ Cerium nitrate significantly reduced the biofilm metabolic activity of *C. albicans*.^27^ The inhibitory activity of CeO_2_ on microbial growth was studied in planktonic cultures and biofilms enumerating the colony‐forming units,^18^ by the agar diffusion method^28^ or by turbidity measurement.^28^

The antiseptic effect of cerium oxide nanoparticles (CeNPs) is still controversial^29^, ^30^, ^31^, ^32^, ^33^, ^34^ with some reports showing no antibacterial activity^30^, ^31^ and others suggesting that CeNPs can exert an antibacterial effect through the oxidative stress of components of the bacteria's cell membrane.^34^ CeNPs have antibacterial activity at sizes below 54 nm on various bacterial strains including *Staphylococcus aureus, E. coli, Pseudomonas aeruginosa, B. subtilis, and Streptococcus pneumoniae*.^35^

Similarly, the current literature on the antibacterial properties of Ce‐BGs is somewhat inconsistent, with some studies reporting a lack of such properties^10^, ^17^, ^36^, ^37^ and others showing microbicidal effects on *E. coli* ^18^, ^19^, ^20^ and *S. aureus*.^38^

In order to contribute to the elucidation of the mechanism underlying the antiseptic properties of these materials, we investigated the antimicrobial activity of Ce‐BGs according to the ISO 22196:2011 method (https://www.iso.org/standard/54431.html) properly adapted and tested against bacteria and fungi.

To this aim, we synthesized BGs, 45S5, and K50S derived glasses, doped with increasing amounts of CeO_2_ (1.2, 3.6, and 5.3 mol%). These BGs have been previously synthesized and thoroughly characterized within our research group.^6^, ^7^, ^8^, ^9^, ^10^, ^11^, ^14^, ^39^, ^40^, ^41^ We then monitored the effect of Ce‐BGs on the pH evolution over time of the medium to assess the role of pH in the antibacterial and antifungal action of the BGs.

## 2 MATERIALS AND METHODS

### 2.1 Cerium‐doped bioactive glasses

The parent glasses are 45S5 and K50S and the molar composition of the studied BGs (hereafter named H0, H1.2, H3.6, H5.3, H series, K0, K1.2, K3.6, K5.3, K series) are presented in Table 1. The samples were prepared as reported^6^ by the melting method and used in the form of slices.^7^ Prior to testing, glass slices of appropriate size were smoothed by lapping.

**TABLE 1:** Nominal composition (mol%) of studied BGs

BG	SiO_2_	Na_2_O	CaO	P_2_O_5_	CeO_2_
H0	46.2	24.3	26.9	2.6	‐
H1.2	45.6	24.0	26.6	2.6	1.2
H3.6	44.5	23.4	26.0	2.5	3.6
H5.3	43.4	23.2	25.7	2.4	5.3
K0	50	25	25	‐	‐
K1.2	49.4	24.7	24.7	‐	1.2
K3.6	48.2	24.1	24.1	‐	3.6
K5.3	47.3	23.7	23.7	‐	5.3

### 2.2 Antimicrobial activity tests

The tests were performed according to the ISO 22196:2011 method, with some adjustments. The Gram‐negative bacteria *E. coli* ATCC 11229 was grown in nutrient broth, *Pseudomonas aeruginosa* ATCC 9027 in tryptic soy broth, the Gram‐positive *Listeria monocytogenes* ATCC 19114 and *S. aureus* ATCC 6538 in brain heart infusion, the yeast *C. albicans* ATCC 10231 in YPD. All the media were provided by BD Difco (Sparks, MD, USA). The cells concentration of fresh overnight cultures was measured by microscope counting in the Thoma's chamber. The culture was properly diluted in 500‐fold diluted culture media to obtain inoculum suspensions of 10^6^ CFU/ml. Where reported, sodium phosphate buffer (PB, pH 7.0) was added at the final concentration of 50 or 200 mM to neutralize the pH increase. For each BG, two slices (with an estimated surface and thickness of about 2.0 cm^2^ and 1 mm, respectively) were placed in a sealed tube and soaked in 0.5 ml of inoculum suspension, the minimum volume necessary for wetting all the surfaces, obtaining an initial concentration of ~2 × 10^5^ CFU/cm^2^. The tubes were incubated for 24 hr at 30°C, then the live microbes were recovered in SCDLP medium and serially diluted in PBS. One liter of SCDLP contained 17 g of casein peptone, 3 g of soybean peptone, 5 g of sodium chloride, 2.5 g of disodium hydrogen phosphate, 2.5 g of glucose, 1 g of lecithin, and 7 g of Tween 80. The appropriate dilutions were spread onto plates of the appropriate medium and colonies were counted after incubation at 30°C for 24 hr for bacteria and for 48 hr for yeasts.

The pH of the soaking solution was monitored for 24 hr after the immersion of the BGs, in the absence of microbial inoculum. The BGs were incubated in 0.5 ml of 500‐fold diluted nutrient broth (NB/500) and in NB/500 supplemented with PB (pH 7.0) at the final concentration of 50 and 200 mM. The pH was discontinuously measured with a pH‐meter equipped with a semi‐micro electrode (XS Instruments, Italy).

## 3 RESULTS AND DISCUSSION

**FIGURE 1:**
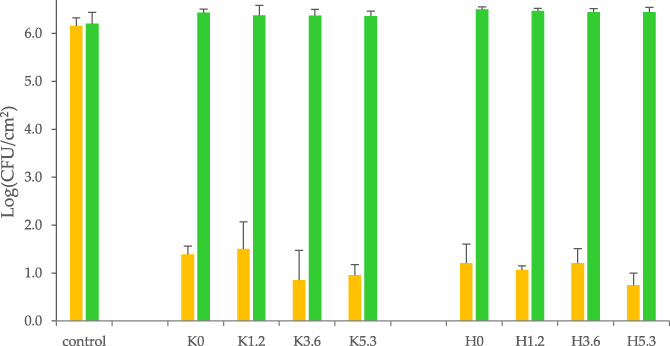
Residual charge of live *Escherichia coli* on the BGs surfaces after 24 hr incubation at 30°C. Inoculum was diluted in NB/500 medium supplemented (green bars) or not (yellow bars) with 200 mM phosphate buffer (pH 7.0). Controls were incubated in the absence of BGs. The reported data (enumeration in LB plates) are means ± standard deviations of three independent experiments

The antimicrobial activity of the Ce‐BGs was first investigated toward the Gram−negative reference strain *E. coli* ATCC 11229. The survival of the strain was determined according to the method ISO 22196:2011 specifically developed to assess the antimicrobial properties of activated surfaces. Slices of Ce‐BGs were immersed in a very diluted medium (NB/500), which provided minimal nutrients for *E. coli* maintenance, containing a bacterial suspension of 1 × 10^6^ CFU/ml, that is, 2 × 10^5^ CFU/cm^2^ of glass surface. After 24 hr of contact with the glasses, a significant reduction of the bacterial charge was detected in all the samples, including those without cerium and regardless of the amount of cerium (Figure 1, yellow bars).

The presence of phosphate in the H series did not affect the extent of inhibition as well, as all the BGs studied showed similar levels of inhibition. As cerium did not seem to confer antibacterial activity per se, a possible explanation for the inhibition of growth observed is the increase of environmental pH, caused by the dissolution of the BGs over time, which is known to exert an antibacterial effect.^28^, ^42^ We then evaluated the influence of pH on the growth of *E. coli* by buffering the medium with 200 mM PB (pH 7.0). In all cases, the growth of *E. coli* in the buffered medium was unaffected by the presence of BGs (Figure 1, green bars), confirming that the antibacterial activity observed was likely due to changes in the pH of the medium rather than to specific effects of the dissolved metal ions.

**FIGURE 2:**
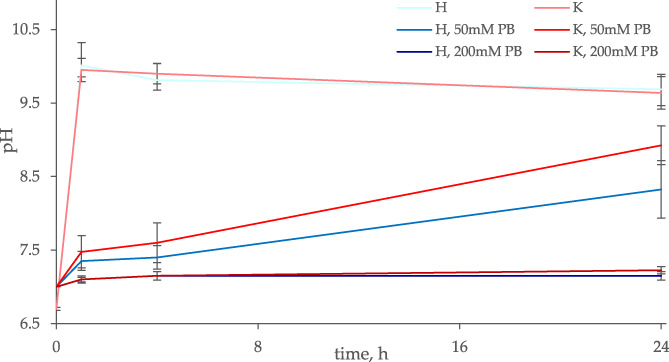
Evolution of pH of the medium NB/500 at different buffering conditions over 24 hr of incubation at 30°C, following the immersion of BGs. H series (blue lines) and K series (red lines) BGs doped with different amounts of cerium oxide (0, 1.2, 3.6, and 5.3 mol%) lead to similar pH values (ANOVA, *p* > .05) and were pooled in unique lines. Increasing concentrations of PB in the medium (0, 50, and 200 mM) correspond to darker colors. The reported data are means ± standard deviations of three independent experiments

We then monitored the evolution of the pH following the immersion of Ce‐BGs and BGs in NB/500 containing different PB concentrations in order to verify whether the contact with BG could affect the pH of the medium at an extent that could hinder microbial vitality (Figure 2). In the absence of PB, the pH increased from 7.0 to 9.9–10.0 in the first hour, lasting to these alkaline values over the next 24 hr. Neither the presence of cerium nor the type of glass did affect the trend of pH (*p* > .05). Conversely, the change in pH was strongly mitigated by the presence of PB in all samples. With 50 mM PB, the increase of pH was slower, reaching 7.4 after 1 hr, 7.5 after 4 hr, 8.4 (H series) and 8.9 (K series) after 24 hr. In the medium supplemented with PB 200 mM, the pH did not change over 24 hr following the immersion of any BGs type, with or without cerium.

A new set of experiments was performed on a wider panel of microorganisms, comprising Gram‐negative *P. aeruginosa*, Gram‐positive *S. aureus* and *L. monocytogenes*, and yeast *C. albicans*, the species most involved in fungal infections. To prevent the change of pH from affecting microbial vitality, the media were buffered at pH 7.0 with 200 mM PB. The presence of cerium did not affect the extent of growth for any microbe (Table 2), even at the highest amount (5.3 mol%).

**TABLE 2:** Charge [Log(CFU/cm^2^)] of microorganisms on the glass surface after 24 hr incubation at 30°C. Inocula were resuspended in the proper medium containing 200 mM PB (pH 7.0). All the Ce‐BGs were tested and compared with controls. The reported data are means ± standard deviations of three independent experiments. No significant difference between the H and K series and among controls and Ce‐BGs with different cerium amounts were observed (test‐t and ANOVA, *p* > .05)

Microorganisms	Control	K series				H series			
		K0	K1.2	K3.6	K5.3	H0	H1.2	H3.6	H5.3
*E. coli*	6.2 ± 0.2	6.4 ± 0.1	6.4 ± 0.2	6.4 ± 0.1	6.4 ± 0.1	6.5 ± 0.1	6.5 ± 0.1	6.4 ± 0.1	6.4 ± 0.1
*P. aeruginosa*	6.8 ± 0.3	7.1 ± 0.2	7.0 ± 0.2	7.0 ± 0.1	6.9 ± 0.1	6.9 ± 0.2	6.9 ± 0.1	6.9 ± 0.1	6.9 ± 0.1
*L. monocytogenes*	6.3 ± 0.4	6.5 ± 0.3	6.5 ± 0.2	6.5 ± 0.1	6.6 ± 0.1	6.7 ± 0.3	6.5 ± 0.1	6.5 ± 0.4	6.6 ± 0.1
*S. aureus*	7.2 ± 0.1	7.0 ± 0.1	7.0 ± 0.2	7.1 ± 0.2	7.2 ± 0.1	7.1 ± 0.2	7.1 ± 0.1	7.1 ± 0.1	7.0 ± 0.2
*C. albicans*	7.0 ± 0.2	7.1 ± 0.1	7.2 ± 0.1	7.2 ± 0.1	7.2 ± 0.2	7.1 ± 0.1	7.3 ± 0.1	7.2 ± 0.1	7.1 ± 0.2

These results confirm on *E. coli* the antimicrobial activity of cerium‐doped 45S5 and K50S already demonstrated against *S. aureus*, a frequent cause of osteomyelitis,^43^ and against other pathogens, such as Gram‐negative bacteria, commonly involved in bone infection.^44^ This notwithstanding, our data strongly suggest that the addition of cerium does not confer a specific antibacterial activity to the BGs investigated.

The effect of pH buffering on the antimicrobial activity of 45S5 and K50S, doped or not with cerium, strongly suggests that hindrance of microbial viability is rather a pH‐related phenomenon. Indeed, bacterial growth inhibition was observed also in the absence of cerium, while buffering the pH near the physiological value eliminated the glass inhibitory effect even in the presence of increasing cerium amount. Interestingly, Allan et al.^28^ already highlighted that 45S5 exhibited an intrinsic antibacterial activity, tested against oral pathogenic bacteria, that was clearly associated with the increase of pH.

The increase of pH of a BG is associated with glass degradation, dissolution, and a spontaneous formation of an apatitic layer.^2^ In vivo, a continuous fluid flow clears the glass dissolution products, thus minimizing changes in the pH. However, alkaline biodegradable materials, when implanted, generate a microenvironmental pH, which is higher than the normal physiological value, reaching up to 9.2.^45^ In the same study, pH dropped to pH 7.7 1 week after implantation, but residual material is expected to influence pH even 9 weeks post‐surgery. The release of alkaline ions drives the nucleation of the apatitic material by raising the local pH, modulates osteoclast cells bone reconstruction, and likely affects bacterial propagation.^45^ In our study, the antibacterial effect seems to be ascribable mainly to the formation of the apatitic layer, without any measurable effect linked to the doping with cerium.

Our results are in accord with some previous literature: when the antibacterial activity of Ce‐BGs was investigated by a zone inhibition method, growth hindrance of *E. coli* and *S. aureus* was similar in Ce‐BGs and in the control.^23^ Also, in all Ce‐BGs studied, the antimicrobial activity seemed mainly due to the composition of native glass, which generated a fast pH increase in the surrounding solution, determining a strong antimicrobial effect, regardless of the addition of cerium. Similar antimicrobial activity against Gram‐positive bacteria, but not against Gram‐negative, was registered by phosphate glass fibers, supplemented or not with cerium,^46^ confirming some intrinsic antimicrobial activity of these BGs not ascribable to cerium doping. In summary, the ability of these Ce‐BGs to hamper the growth of pathogens remains, and it is generally recognized in vivo, albeit it cannot be directly ascribed to the presence of cerium ions.

## 4 CONCLUSIONS

Cerium‐doped bioactive glasses are promising biomaterials that present low toxicity to normal cells, modulate reactive oxygen species levels, and were confirmed to exert a considerable antibacterial effect. This notwithstanding, this effect is not directly associated with the presence of cerium, at least up to a 5.3 mol% content. We have previously shown that higher cerium amounts in the BG composition lead to the formation of ceramic and not vitreous material. An increase in cerium content is then not a viable strategy to achieve cerium‐based materials with both antioxidant and antibacterial properties. To this end, we postulate that alternative approaches such as doping with additional TII or functionalization with drugs should be considered.

## CONFLICTS OF INTEREST

The authors declare no conflict of interest.

## Data Availability

All experimental data and discussed results of this technical note are available.
